# Precarious employment in young adulthood and later alcohol-related morbidity: a register-based cohort study

**DOI:** 10.1136/oemed-2023-109315

**Published:** 2024-04-16

**Authors:** Emelie Thern, Devy L Elling, Kathryn Badarin, Julio César Hernando Rodríguez, Theo Bodin

**Affiliations:** 1 Institute of Environmental Medicine (IMM), Karolinska Institute, Stockholm, Sweden; 2 Department of Public Health Sciences, Stockholm University, Stockholm, Sweden; 3 Centre for Occupational and Environmental Medicine, Stockholm, Sweden

**Keywords:** precarious employment, young adults, alcohol, register-based, cohort

## Abstract

**Objectives:**

The prevalence of precarious employment is increasing, particularly among young adults where less is known about the long-term health consequences. The present study aims to test if being precariously employed in young adulthood is associated with an increased risk of alcohol-related morbidity later in life.

**Methods:**

A register-based cohort study was conducted in Sweden. The Swedish Work, Illness, and Labor-market Participation (SWIP) cohort was used to identify individuals who were aged 27 years between 2000 and 2003 (*n*=339 403). Information on labour market position (precarious employment, long-term unemployment, substandard employment and standard employment relations) was collected for young people 3 years after graduation from school using nationwide registers. Details about alcohol-related morbidity during a 28-year follow-up period were collected from the National Hospital Discharge Register. Data on sex, age, country of birth, education and previous poor health were also obtained from the registers.

**Results:**

Young adults in precarious employment had an increased risk of alcohol-related morbidity compared with individuals of the same age in standard employment (HR 1.43, 95% CI 1.32 to 1.55), after adjusting for several important covariates. A stronger association was found among young men who were precariously employed compared with young women.

**Conclusion:**

This nationwide register-based study conducted in Sweden with a long-term follow-up suggests that being precariously employed in young adulthood is associated with an increased risk of alcohol-related morbidity later in life.

**WHAT IS ALREADY KNOWN ON THIS TOPIC**

- Precarious employment is a known social determinant of health; previous research has shown that being precariously employed in young adulthood is associated with an increased risk of mental health problems and greater self-reported alcohol use.

**WHAT THIS STUDY ADDS**

- The results of this study suggest that being precariously employed in young adulthood is associated with an increased risk of alcohol-related morbidity requiring inpatient care.

**HOW THIS STUDY MIGHT AFFECT RESEARCH, PRACTICE OR POLICY**

- These finding have implications for the discussion on ‘if any job is really better than no job at all’ given that the risk estimates for young adults who were precariously employed were only marginally less than the results for the long-term unemployed. Our results strengthen the notion of conceptualising employment status as a continuum rather than dichotomising status into employed or unemployed.

## Background

The increase in non-standard work arrangements has reduced the boundaries between employment and unemployment. As a result, there needs to be a shift in focus from the consequences of being completely excluded from the labour market to the consequences of having a weaker and/or less secure attachment to the labour market. Employment conditions, including precarious employment, are an important social determinant for health and health inequalities.^1 2^ Precarious employment is less secure and often comes with fewer benefits compared with more standard work arrangements. The disadvantages can include lack of employment contractual security (ie, temporary employment), low wages, economic hardship and limited social protection and workplace rights.^1 3^ In addition, alcohol-related health problems are significant public health issues because they can have a negative impact on overall health for individuals throughout all stages of life from childhood to adulthood.^4^ These issues can also lead to decreased workplace productivity and increased healthcare costs for society,^4–6^ which is relevant for the already vulnerable groups in the population who are disproportionately affected by precarious employment, such as youths, young adults and women.^7–10^ Nevertheless, the long-term alcohol-related health consequences of being in precarious employment are not yet fully understood.

‘Emerging adulthood’ is a particular life stage that describes a young person’s transition into the labour market, which could be considered a difficult and sensitive time.^11^ Young adults (15–29 years of age) trying to establish themselves in the labour market after finishing school are particularly vulnerable because they lack work experience, work opportunities and social security benefits in the case of unemployment. The high unemployment rate that started during the 1990s in Sweden and the increased prevalence of precarious employment have hit young adults disproportionally hard compared with other age groups.^9 10^ Furthermore, evidence suggests that failure to successfully establish oneself in the labour market can have long-term adverse health consequences.^8 10 12–15^

Being precariously employed could be considered a stressful event with lower income, feelings of unfairness and greater uncertainty about future employment prospects, which could increase the risk of mental health problems.^15^ Previous research on the general working population suggests that women appear to be more vulnerable to the mental health effects of precarious employment than men.^16^ This could be due to the gendered nature of the labour market and household work. However, this trend does not seem to hold true for young adults.^13 17^ A systematic review has suggested that being precariously employed could trigger unhealthy coping strategies, such as an increase in alcohol consumption.^18^ Although a decrease in alcohol consumption has been observed among Swedish youths during recent decades, the majority of young adults consume alcohol.^19^ Furthermore, having a lower income could result in social and material deprivation, which could indirectly affect other social determinants of health (eg, adverse lifestyles).^1 18 20^ Youth unemployment has been linked to alcohol misuse and alcohol-related harm.^6 21–23^ However, only a few studies have investigated the association between precarious employment, alcohol consumption and alcohol-related harm.^24 25^ A recent study on the whole Swedish working population found an increased risk of alcohol use disorders, requiring either specialised or inpatient care, among precariously employed workers.^24^ A Korean study suggested that persistent precarious employment in young adulthood is associated with an increased risk of self-reported alcohol use problems (using the CAGE questionnaire), especially among young men.^25^

Young people in Sweden tend to report a higher prevalence of alcohol intake and related harm compared with the general population.^4 19^ This makes it challenging to distinguish between normal behaviour and clinically significant aspects of alcohol misuse when relying solely on self-reported information about alcohol consumption.^25^ One way to overcome this limitation in previous research is to use healthcare data to measure alcohol-related harm. Given that these diagnoses generally represent alcohol-related harm from serious alcohol abuse over several years, they are less likely to solely reflect the normative behaviour of young people.^26^

This paper aims to investigate whether being precariously employed in young adulthood is associated with an increased risk of alcohol-related morbidity later in life. We obtained data from high-quality nationwide registers and followed a large cohort of young adults during a 28-year follow-up.

## Methods

### Study population

The study population included individuals in the Swedish Work, Illness, and Labour Market Participation (SWIP) cohort who were born between 1973 and 1976. The SWIP cohort was created by linking several nationwide registers for individuals aged 16–65 years who were registered as a resident in Sweden in 2005. More information about the cohort can be found elsewhere.^27^ As this study focused on labour market position at a young age, the cut-off age of 27 years was chosen in order to collect exposure information, which is measured 3 years after graduation, before the age of 30 years.^13 15^

**Figure 1:**
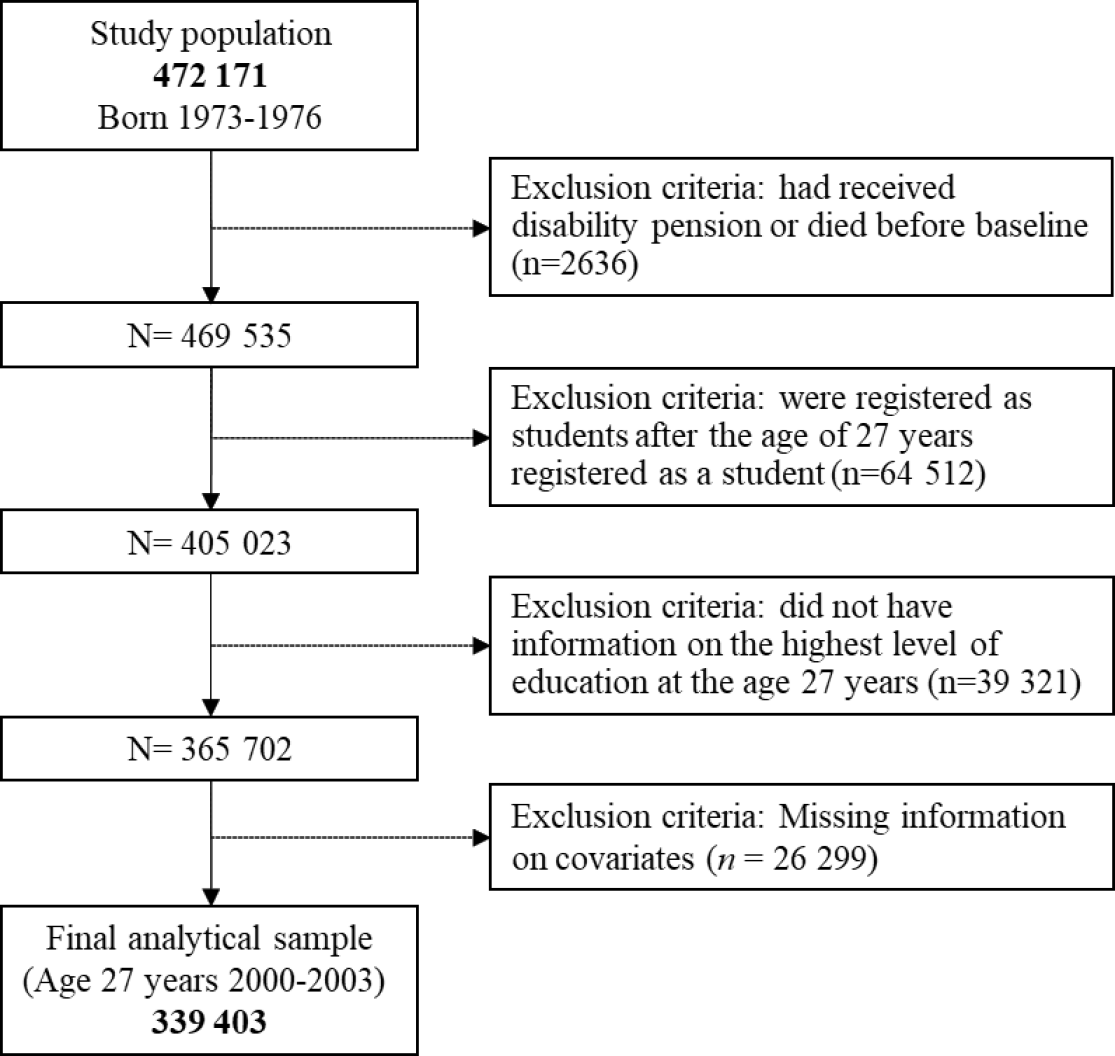
Flowchart of selection process of analytical sample.

Individuals were excluded if they (1) had received disability pension or died before baseline (3 years after graduation from school), (2) were registered as a student after the age of 27 years, (3) did not have information on the highest level of education at the age 27 years, or (4) had missing information on covariates (figure 1).

A total of 339 403 individuals were included in the final analytical sample. Compared with the analytical sample, a higher proportion of the excluded individuals were female, born outside of Sweden, and had parents with lower levels of educational attainment and socioeconomic status (SES) (online supplemental Table S1).

10.1136/oemed-2023-109315.supp2 Supplementary data

### Exposure: labour market position in young adulthood

Information on labour market positions was collected from the registers 3 years after finishing the highest level of education (primary, secondary or university). Three years after graduation was originally selected as it has been deemed to be the most suitable time point to determine the end of the school-to-work transition.^28^

The labour market position among the study participants was assessed in two steps using the Longitudinal Integration Database for Health Insurance and Labour Market Studies (LISA) register.^13 15^ During the first step, information on the year of examination from school and the level of education (primary, secondary or university) was obtained the year the participants turned 27 years old. Participants in the current study graduated between 1989 and 2003 when they were aged 16–27 years. Around 7.8% of the analytical sample had missing information on the year of examination; therefore, for these individuals, a crude measure of the year of examination was calculated using the level of education of each birth cohort. In the second step, we used the year of examination and added 3 years to pinpoint the baseline, which was when we obtained information on labour market position. In other words, information on labour market position was obtained between 1992 and 2006 when the participants were aged 19–30 years. Consequently, participants had a different start for follow-up time depending on when they graduated from school (see online supplemental figure 1 for more details).

10.1136/oemed-2023-109315.supp1 Supplementary data

The study population was categorised into five mutually exclusive groups in the following order: precarious employment (PER), long-term unemployed, substandard employment relations (SSER), standard employment relations (SER), and others. The reference category was SER.

The Swedish Register-based Operationalization of Precarious Employment (SWE-ROPE) version 2.0 was used to define PER, SSER and SER.^29^ The SWE-ROPE includes five different components (contractual employment insecurity, temporariness, multiple-job holding, income level, and coverage under collective bargaining agreement) to encompass the three main dimensions of PER that had been identified by previous research (employment insecurity, income inadequacy, and lack of rights and protection), as seen in table 1.^3 30^ Using the LISA register, each of these five components was individually scored, as well as summed up to a total index ranging from −9 to 2.^30^ Previous established cut-off points for PER, SSER and SER^31^ were used in the current study. Individuals scoring less than −3 were classified as PER, individuals scoring between −3 and −1 were classified as SSER, and individuals scoring ≥0 were classified as SER.

**Table 1:** Swedish Register-based Operationalization of Precarious Employment (SWE-ROPE) scoring of items

Item	Score				
	−2	−1	0	1	2
Contractual employment insecurity		Agency employed	Directly employed		
Temporariness	Unstable employment		Stable employment		
Multiple-job holding	Multiple jobs (>2 jobs) and multiple sectors (>1 sector)	Multiple jobs (>2 jobs)	No multiple jobs (one job)		
Income level (% of median)	<60	60–80	81–120	121–200	>200
Coverage under collective bargaining agreement (% likelihood)	<70	70–90	91–100		

Long-term unemployment was defined as being unemployed for at least 180 days for 1 year (3 years after graduation). The last group of ‘other’ included everyone who was not classified into any of the other groups (eg, self-employed, not registered as employed, or student). This group was created to reduce the potential issue of selection bias that can arise when excluding people.

### Outcome: alcohol-related morbidity

Information on the outcome of alcohol-related morbidity requiring inpatient care was obtained from the National Hospital Discharge Register using the Swedish versions of the International Statistical Classification of Disease (ICD) version 9 (1987–1996) and version 10 (from 1997). The following diagnoses were included in the outcome: mental and behavioural disorders due to alcohol (ICD-9: 291, 303; ICD-10: F10), alcoholic liver disease (ICD-9: 571; ICD-10: K70), and toxic effect of alcohol (ICD-9: 980; ICD-10: T51).^32^ During follow-up, participants with a first-time admission for any of the listed alcohol-related diagnoses, either as a principal or contributory discharge of diagnoses, was considered as having the outcome of interest.

### Covariates

The selection and inclusion of covariates was based on previous research.^10 14 15^ Sociodemographic information on the study population such as sex, birth year, country of birth (Sweden or outside of Sweden), age (continuous), own highest level of education (primary, secondary or university) was collected from the LISA register at baseline. Information on the highest level of education (primary, secondary or university) and SES (non-manual, manual, self-employed/farmer and not classified) of either parent was collected from the Statistics Sweden’s Multi-Generation Register (MGR) and the LISA register.

From the National Hospital Discharge Register, we extracted information on any previous mental health problems requiring inpatient care (ICD-9: 291–319; ICD-10: F00–F99) and alcohol-related health problems (using the same diagnosis codes as the outcome) before the start of the follow-up period. The National Hospital Discharge Register was launched in 1973 for psychiatric diagnoses and had reached complete national coverage by 1987. A measure of any parent’s alcohol-related health problems, including alcohol-related morbidity (same ICD codes as the outcome) and alcohol-related mortality (same as outcome and additional ICD-10 codes, covering alcohol diseases of the nervous system, circulatory system and digestive system, including E24.4, G31.2, G62.1, G72.1, I42.6, K29.2, K85.2, K86.0, O35.4, R78.0, Z04.0, Z71.4, Z72.1, and the corresponding ICD-9 codes) was included. The Cause of Death Register was established in 1961 in Sweden.

More detailed information on alcohol-related diagnoses was available in the Cause of Death Register; subsequently, we were able to include more diagnoses concerning parents’ alcohol-related health problems. The covariates were categorised as indicated in table 2.

**Table 2:** Baseline characteristics of study population, stratified by labour market position in young adulthood

	Precarious employment, N (%)	Long-term unemployed, N (%)	Substandard employment relation, N (%)	Standard employment relation, N (%)	Other, N (%)	P value
Total	42 232 (12.4)	26 476 (7.8)	107 395 (31.6)	133 141 (39.2)	30 159 (8.9)	
Sex						<0.001
Male	21 761 (51.5)	15 571 (58.8)	56 132 (52.3)	71 079 (53.4)	15 266 (50.6)	
Female	20 471 (48.5)	10 905 (41.2)	51 263 (47.7)	62 062 (46.6)	14 893 (49.4)	
Country of birth						<0.001
Sweden	39 889 (94.5)	24 420 (92.2)	103 504 (96.4)	129 362 (97.2)	26 645 (88.4)	
Outside of Sweden	2343 (5.6)	2056 (7.8)	3891 (3.6)	3779 (2.8)	3514 (11.7)	
Birth year						<0.001
1973	7762 (18.4)	8762 (33.1)	26 310 (24.5)	38 884 (29.2)	7648 (25.4)	
1974	10 081 (23.9)	8074 (30.5)	27 531 (26.5)	35 272 (26.5)	8429 (28.0)	
1975	11 518 (27.3)	5986 (22.6)	26 726 (24.9)	31 047 (23.3)	7373 (24.5)	
1976	12 871 (30.5)	3654 (25.0)	26 828 (25.0)	27 938 (21.0)	6709 (22.3)	
Age at baseline (mean±SD)	23.6±3.2	22.7±3.1	24.3±3.6	24.8±3.5	22.8±3.6	
Education						<0.001
Primary	3285 (7.8)	3718 (14.0)	5031 (4.7)	6772 (5.1)	7546 (25.0)	
Secondary	27 761 (65.7)	18 261 (69.0)	60 479 (56.3)	60 248 (45.3)	15 919 (52.8)	
University	11 186 (26.5)	4497 (17.0)	41 885 (39.0)	66 121 (50.0)	6694 (22.2)	
Previous mental health problems	1397 (3.3)	1095 (4.1)	2408 (2.2)	2397 (1.8)	2511 (8.3)	<0.001
Previous alcohol-related health problems	141 (0.3)	78 (0.3)	270 (0.3)	238 (0.2)	135 (0.5)	<0.001
Parental education						<0.001
Primary	6346 (15.0)	5497 (20.8)	15 700 (14.6)	18 909 (14.2)	6431 (21.3)	
Secondary	21 225 (50.3)	14 544 (54.9)	54 325 (50.6)	65 179 (49.0)	14 636 (48.5)	
University	14 661 (34.7)	6435 (24.3)	37 370 (34.8)	49 053 (36.8)	9092 (30.2)	
Parental socioeconomic status						<0.001
Non-manual	22 176 (52.5)	10 750 (40.6)	59 374 (55.3)	77 301 (58.1)	12 873 (42.7)	
Manual	14 694 (34.8)	11 828 (44.7)	37 109 (34.6)	44 854 (33.7)	11 129 (36.9)	
Self-employed/farmer	1924 (4.6)	952 (3.6)	4838 (4.5)	5009 (3.8)	1468 (4.9)	
Not classified	3438 (8.1)	2946 (11.1)	6074 (5.7)	5978 (4.5)	4689 (15.6)	
Parents’ alcohol-related health problems	1817 (4.3)	1607 (6.1)	3051 (2.8)	3127 (2.4)	2118 (7.0)	<0.001
Outcome						
Alcohol-related morbidity	986 (2.3)	1065 (4.0)	1720 (1.6)	1702 (1.3)	1240 (4.1)	<0.001

Other: self-employed, not registered as employed, or student.

### Statistical analysis

Pearson’s χ^2^ test was used to determine differences in baseline characteristics between groups. The association between labour market position 3 years after graduation from school and later alcohol-related morbidity was estimated by Cox regression analyses to obtain hazard ratios (HRs) with 95% confidence intervals (CIs). Schoenfeld residuals were used to test the proportional hazard assumption and found not to be violated (p-value≥0.05). Person-time was calculated from 4 years after graduation from school (1 January 1993, at the earliest), until the first date of alcohol-related diagnoses, date of emigration, date of death, or end of follow-up (31 December 2020), whichever came first.

In the regression analyses, a crude model with no adjustments was first fitted. Then, all the individual-level covariates (as listed in table 2, except for previous mental health and alcohol-related health problems) were entered into the first model. Previous mental health and alcohol-related health problems were of particular interest due to the issue of health selection (ie, poor health is a risk factor for precarious employment), thus this information was added separately in model 2. In the final model, family level covariates (highest level of either parents’ education and SES, as well as parents’ alcohol-related health problems) were also included as potential confounders.

Given that previous research suggests there could be sex differences in the vulnerability to the effects of precarious employment and the outcome of harmful alcohol varies between men and women, the main analyses were also stratified by sex.^16 19^

To further reduce bias due to health selection, sensitivity analyses were conducted excluding all individuals with previous mental health or alcohol-related health problems. Individuals in PER with short-term unemployment (less than 180 days) were recategorised into the group ‘other’ to assess if the increased risk of alcohol-related morbidity could be explained by experiences of unemployment, which has been found in previous research.^23^ All analyses were performed using Stata Statistical Software, release 17.

## Results

### Baseline characteristics

Baseline characteristics stratified by labour market establishment 3 years after graduation are presented in table 2. Most of the individuals were categorised to be in SSER or SER, while 12.4% were categorised as being in PER. In general, individuals categorised as PER were male with lower levels of education compared with individuals in SER. Previous mental health and alcohol-related health problems were to a greater extent reported by individuals categorised as long-term unemployed and other. Parents had a similar level of education among individuals in SER and PER. A lower prevalence of parents in non-manual occupations was found among young adults in PER compared to individuals in SER.

A total of 6713 (2.0%) individuals required inpatient care at least once for alcohol-related morbidity during a maximum 28-year follow-up (table 2), of which 4448 (66.6%) were male and 2265 (33.7%) were female. The mean follow-up time was 21.0 years for men and 20.5 years for women.

### Alcohol-related morbidity

In the crude analyses for the total population, compared with young adults in SER, almost a twofold increased risk of alcohol-related morbidity was found among young adults in PER (table 3). Young adults in long-term unemployment had almost a threefold increased risk. Adjusting for several important covariates, including previous mental health and alcohol-related health problems, attenuated the risk substantially but the increased risk remained, especially among young adults in PER (HR 1.43, 95% CI 1.32 to 1.55) and in long-term unemployment (HR 1.95, 95% CI 1.80 to 2.11). A slight increased risk was also found among young adults in SSER, after adjusting for all covariates (HR 1.15, 95% CI 1.07 to 1.23).

**Table 3:** Crude and adjusted hazard ratios (HRs) with 95% confidence intervals (CIs) for the association between labour market position in young adulthood and later alcohol-related morbidity, for total cohort and stratified by sex

	Crude HR (95%CI)	Model 1 HR adjusted (95%CI)	Model 2 HR adjusted (95%CI)	Model 3 HR adjusted (95%CI)	Number of events (%)
Total					
SER (reference)	1.00	1.00	1.00	1.00	1702 (1.3)
PER	1.77 (1.64 to 1.92)	1.47 (1.36 to 1.60)	1.42 (1.31 to 1.54)	1.43 (1.32 to 1.55)	986 (2.3)
Long-term unemployed	2.71 (2.51 to 2.93)	2.11 (1.95 to 2.28)	2.00 (1.85 to 2.16)	1.95 (1.80 to 2.11)	1065 (4.0)
SSER	1.24 (1.16 to 1.32)	1.16 (1.09 to 1.24)	1.15 (1.07 to 1.23)	1.15 (1.07 to 1.23)	1720 (1.6)
Other	2.88 (2.67 to 3.10)	1.97 (1.83 to 2.13)	1.65 (1.53 to 1.79)	1.64 (1.51 to 1.77)	1240 (4.1)
Male					
SER (reference)	1.00	1.00	1.00	1.00	1147 (1.6)
PER	1.82 (1.65 to 2.01)	1.53 (1.39 to 1.69)	1.48 (1.34 to 1.63)	1.50 (1.36 to 1.65)	653 (3.0)
Long-term unemployed	2.68 (2.44 to 2.94)	2.21 (2.01 to 2.43)	2.10 (1.91 to 2.31)	2.04 (1.86 to 2.25)	752 (4.8)
SSER	1.24 (1.15 to 1.35)	1.17 (1.08 to 1.27)	1.16 (1.06 to 1.26)	1.16 (1.07 to 1.26)	1149 (2.1)
Other	2.77 (2.53 to 3.04)	1.95 (1.77 to 2.15)	1.66 (1.51 to 1.84)	1.66 (1.50 to 1.83)	747 (4.9)
Female					
SER (reference)	1.00	1.00	1.00	1.00	555 (0.9)
PER	1.74 (1.52 to 2.00)	1.36 (1.18 to 1.56)	1.31 (1.14 to 1.51)	1.32 (1.14 to 1.51)	333 (1.6)
Long-term unemployed	2.60 (2.27 to 2.99)	1.88 (1.63 to 2.17)	1.78 (1.55 to 2.05)	1.75 (1.52 to 2.02)	313 (2.9)
SSER	1.24 (1.10 to 1.39)	1.13 (1.01 to 1.28)	1.12 (1.00 to 1.26)	1.12 (1.00 to 1.26)	571 (1.1)
Other	3.18 (2.82 to 3.60)	1.98 (1.74 to 2.50)	1.61 (1.41 to 1.84)	1.59 (1.39 to 1.82)	493 (3.3)

Model 1: Adjusted for sex (total only), country of birth, year of birth, age at baseline, highest levels of own education.

Model 2: Additional adjustment for previous mental health problems and previous alcohol-related health problems requiring inpatient care.

Model 3: Additional adjustment for highest level of parents’ educational attainment and socioeconomic status, as well as parents’ alcohol-related health problems.

Other: self-employed, not registered as employed or student.

PER, precarious employment relation; SER, standard employment relation; SSER, sub-standard employment relation.

In the analyses stratified by sex, the association between PER and alcohol-related morbidity appears to be stronger among men (HR 1.50, 95% CI 1.36 to 1.65) compared with women (HR 1.32, 95% CI 1.14 to 1.51).

### Sensitivity analyses

Excluding individuals with previous mental health and alcohol-related health problems (*n*=10 716) demonstrated a similar, slightly attenuated, association between PER and alcohol-related morbidity in the total population (as seen in online supplemental table 2). Furthermore, additional analyses recategorising precariously employed individuals with unemployment (*n*=25 718) into the group of ‘other’ demonstrated a slightly attenuated positive association between PER and alcohol-related morbidity (online supplemental table 3). In the last sensitivity analyses, we did not see any large effects on the risk estimates when excluding individuals with missing information on the year of examination (*n*=24 512) (online supplemental table 4).

## Discussion

The results of this register-based cohort study suggest that being precariously employed in young adulthood is associated with an increased risk of alcohol-related morbidity later in life, especially among young men but also among young women.

These results support and build on the current literature on the health consequences of precarious employment among young adults.^8 10 14 15^ We found a similar increased risk of later alcohol-related morbidity among precariously employed young adults, as shown in an earlier study on the effects of precarious employment in the general working-age population.^24^ Extending previous research on the positive association between precarious employment in young adulthood and self-reported alcohol use problems,^25^ the results of the current study suggest an increased risk for alcohol-related morbidity requiring inpatient care. Another important difference between this study and the study on self-reported alcohol use was the measure of precarious employment. A previous study investigated persistent precarious employment, concluding the longer the precarious employment, the higher the risk of alcohol use problems.^25^ However, this topic was not the focus of the current study.

A potential explanation for our findings could be that young adulthood is a particularly sensitive period in life concerning the initiation and formation of health-related behaviours, such as alcohol use. Subsequently, young adults in precarious employment might use alcohol as a coping strategy for stress and form a habit of consuming larger quantities of alcohol compared with individuals in SER of the same age. This habit might not change when and if they move into more stable and higher-quality employment. In line with previous research, we found that young men tend to be more vulnerable to precarious employment compared with young women.^25^ However, previous research also suggests that women tend to use alcohol to a greater extent to deal with stress compared with men.^33^ This finding suggests that our results are more likely driven by other mechanisms, something that is beyond the scope of the current study.

Alcohol misuse has also been shown to be a risk factor for later labour market marginalisation in terms of increased risk of unemployment, sickness absence and disability pension.^34^ In our sensitivity analyses we found that excluded participants (a diverse group including individuals with a disability pension and mostly students over 27) had to a greater extent previous alcohol-related health problems requiring inpatient care compared with the included participants. However, we did not examine whether this group is more likely to face marginalisation in the future as it was beyond the scope of our study. Furthermore, adjusting and excluding individuals with previous mental health and alcohol-related health problems demonstrated a slightly attenuated effect of precarious employment, suggesting that there could be some health selection in precarious employment.

As expected, and in line with previous research,^23^ we found an increased risk of alcohol-related morbidity among young adults in long-term unemployment 3 years after graduating from school. Being unemployed could be considered a critical life event illustrated by the loss of income, social status, social network, important labour experience and identity. It should be acknowledged that in the current study, we only captured individuals who receive unemployment benefits. Consequently, the association might have been stronger if we were able to also include the unemployed population without benefits. To deal with the increased stress of being unemployed, unhealthy coping strategies might be initiated, such as increased alcohol consumption, which in turn affects the likelihood of securing employment.^6 35^ To escape unemployment, several young people take the first job offered, which is generally more precarious with less security, lower wages and longer hours. The results of the current study suggest that this decision appears to be marginally more beneficial compared with remaining unemployed, which adds to the discussion of whether any job is really better than no job at all.^36^

### Strengths and limitations

Register-driven data with long-term follow-up and a large sample size are all major strengths of this study. The SWIP cohort includes information from several nationwide registers for the index person, as well as their parents. A limitation of the SWIP cohort in relation to the current study is that it only includes individuals registered in Sweden in 2005; subsequently, everyone born between 1973 and 1976 who died before 2005 was excluded due to left truncation in the registers. Using a separate database, we have previously calculated that this register constraint should have marginal effects on the estimate as only 1.5% of the birth cohorts (1973–1976) died before 2005. The Swedish registers have limited coverage of educational attainment outside of Sweden. As a result, many individuals born outside of Sweden were excluded from the study due to missing information on their parents’ educational attainment. This might impact the generalisability of the results.

Information on the exposure of labour market position in young adulthood was obtained from nationwide registers, decreasing the issue of recall bias and attrition. Using the SWE-ROPE to define precarious employment is a strength as it uses a multidimensional construct instead of only one dimension, reducing the risk of misclassification.^37 38^ A limitation of the SWE-ROPE is that it does not include other important dimensions of precarious employment, such as rights, perspective and working hours (part-time or full-time).^37^ Information on the exposure was collected during a period of high unemployment in Sweden from many of the individuals included in the current study, which could have influenced the results. During the beginning of the 1990s, Sweden along with many other countries was hit by the economic crises, which increased unemployment drastically, especially among youth and young adults.^39^ To tackle the increase in unemployment, many initiatives, including schemes to prolong education, were implemented resulting in more individuals staying in school longer, which was especially evident among young women.^39^ Consequently, a large proportion of the individuals excluded were female who were registered as students after the age of 27 years, which could have biased the results. Another limitation is that given that individuals complete their education at different ages, follow-up periods vary. This could lead to uneven contextual influences (eg, high unemployment in the 1990s) on participants’ labour market participation that could affect the results to some extent.

Another strength of this study is the use of the Hospital Discharge Register to collect information on the outcome of alcohol-related morbidity; as the outcome is well defined, it decreases self-reported bias and social desirability issues. This is especially important for the population of young adults because young people tend to report a higher prevalence of alcohol consumption and related harms compared with the general population, where such behaviours are often underreported.^26^ A limitation of the Hospital Discharge Register is that it only captures the more severe cases of alcohol-related health problems. The inclusion of previous mental health and alcohol-related health problems as well as information on parents’ health is a strength,^22^ although with the same limitations as previously described.

## Conclusion

This nationwide register-based study conducted in Sweden with a 28-year follow-up suggests that being precariously employed in young adulthood is associated with an increased risk of alcohol-related morbidity later in life. This is important as young adults are especially vulnerable due to the increase in the prevalence of precarious employment.

## Data Availability

Data may be obtained from a third party and are not publicly available.
