# Supplementary material for: Precarious employment in young adulthood and later alcohol-related morbidity: a register-based cohort study
Source: Occup Environ Med. 2024 Apr 16;81(4):201–8. doi: 10.1136/oemed-2023-109315 (PMC11103336; doi:10.1136/oemed-2023-109315)
Supplement: Supplementary data [file oemed-2023-109315supp002.pdf]

Supplementary table 1. Baseline characteristics included and excluded individuals

	Included<br>%	Excluded<br>%	P- value
Total	71.9	28.1	
Sex			<0.001
Male	53.0	45.5	
Female	47.0	54.5	
Country of birth			<0.001
Sweden	95.4	51.5	
Outside of Sweden	4.6	48.5	
Birth year			<0.001
1973	26.3	24.3	
1974	26.3	25.0	
1975	24.4	25.6	
1976	23.0	25.1	
Age at baseline (mean±SD)	24.2 ± 3.5	24.8 ± 3.7	
Education			<0.001
Primary	7.8	10.0	
Secondary	53.8	34.8	
University	38.4	32.7	

Missing	0	22.6	
Prior mental health problems	2.9	3.0	0.201
Prior alcohol-related health problems	0.3	0.5	<0.001
Parental education			<0.001
Primary	15.6	6.2	
Secondary	50.1	21.3	
University	34.4	21.6	
Missing	0	51.0	
Parental SES			<0.001
Non-manual	53.8	31.4	
Manual	35.2	16.0	
Self-employed/farmer	4.2	1.9	
Not classified	6.8	50.7	

SES: socioeconomic status

Thern E, *et al. Occup Environ Med* 2024; 81:201–208. doi: 10.1136/oemed-2023-109315

BMJ Publishing Group Limited (BMJ) disclaims all liability and responsibility arising from any reliance placed on this supplemental material which has been supplied by the author(s)

Supplementary Table 2. Crude and adjusted HRs and 95% CI for the association between labour market position in young adulthood and later alcohol-related morbidity, excluding individuals with previous mental and alcohol-related health problems (n =10 176).

	PER<br>HR (95%CI)	Long-term unemployed<br>HR (95%CI)	SSER<br>HR (95%CI)	SER<br>HR (95%CI)	Other<br>HR (95%CI)
Crude	1.62 (1.49, 1.77)	2.55 (2.35, 2.77)	1.19 (1.11, 1.27)	1.00	2.48 (1.11, 1.27)
Model 1	1.37 (1.30, 1.49)	2.00 (1.84, 2.18)	1.12 (1.04, 1.20)	1.00	1.78 (1.64, 1.94)
Model 2	1.38 (1.27, 1.50)	1.96 (1.80, 2.13)	1.12 (1.05, 1.21)	1.00	1.76 (1.62, 1.92)

PER: Precarious employment relation, SSER: sub-standard employment relation, SER: standard employment relation, Other: self-employed, not registered as employed, or student

Model 1: Adjusted for sex, country of birth, year of birth, age at baseline, highest levels of own education.

Model 2: Additional adjustment for highest level of parents' educational attainment and socioeconomic status.

Thern E, *et al. Occup Environ Med* 2024; 81:201–208. doi: 10.1136/oemed-2023-109315

Supplementary Table 3. Crude and adjusted HRs and 95% CI for the association between labour market position in young adulthood and later alcohol-related morbidity, re-categorized individuals with any unemployment from the precarious employment group (n= 25 718) into the group 'other'

	PER<br>HR (95%CI)	Long-term unemployed<br>HR (95%CI)	SSER<br>HR (95%CI)	SER<br>HR (95%CI)	Other<br>HR (95%CI)
Crude	1.42 (1.26, 1.61)	2.71 (2.51, 2.92)	1.24 (1.16, 1.32)	1.00	2.47 (2.32, 2.64)
Model 1	1.29 (1.12, 1.45)	2.10 (1.94, 2.27)	1.17 (1.09, 1.25)	1.00	1.80 (1.68, 1.93)
Model 2	1.23 (1.08, 1.39)	2.00 (1.85, 2.16)	1.15 (1.07, 1.23)	1.00	1.60 (1.49, 1.71)
Model 3	1.25 (1.10, 1.42)	1.95 (1.80, 2.11)	1.15 (1.07, 1.23)	1.00	1.60 (1.49, 1.71)

PER: Precarious employment relation, SSER: sub-standard employment relation, SER: standard employment relation, Other: self-employed, not registered as employed, or student

Model 1: Adjusted for sex, country of birth, year of birth, age at baseline, highest levels of own education.

Model 2: Additional adjustment for prior mental health and alcohol-related health problems requiring inpatient care.

Model 3: Additional adjustment for highest level of parents' educational attainment and socioeconomic status.

Thern E, *et al. Occup Environ Med* 2024; 81:201–208. doi: 10.1136/oemed-2023-109315

Supplementary Table 4. Crude and adjusted HRs and 95% CI for the association between labour market establishment at a young age and later alcohol-related morbidity, excluding individuals with missing information on the year of exam (n =24 512).

	PER<br>HR (95%CI)	Long-term unemployed<br>HR (95%CI)	SSER<br>HR (95%CI)	SER<br>HR (95%CI)	Other<br>HR (95%CI)
Crude	1.42 (1.24, 1.62)	2.65 (2.44, 2.88)	1.28 (1.19, 1.37)	1.00	2.45 (2.28, 2.63)
Model 1	1.27 (1.11, 1.45)	2.11 (1.94, 2.29)	1.19 (1.11, 1.28)	1.00	1.84 (1.71, 1.98)
Model 2	1.22 (1.07, 1.40)	1.99 (1.83, 2.16)	1.18 (1.10, 1.26)	1.00	1.68 (1.56, 1.81)
Model 3	1.24 (1.09, 1.42)	1.94 (1.78, 2.11)	1.18 (1.10, 1.27)	1.00	1.67 (1.55, 1.80)

PER: Precarious employment relation, SSER: sub-standard employment relation, SER: standard employment relation, Other: self-employed, not registered as employed, or student

Model 1: Adjusted for sex, country of birth, year of birth, age at baseline, highest levels of own education.

Model 2: Additional adjustment for prior mental health and alcohol-related health problems requiring inpatient care.

Model 3: Additional adjustment for highest level of parents' educational attainment and socioeconomic status.

Thern E, *et al. Occup Environ Med* 2024; 81:201–208. doi: 10.1136/oemed-2023-109315
