# Supplementary material for: Precarious employment in young adulthood and later alcohol-related morbidity: a register-based cohort study
Source: Occup Environ Med. 2024 Apr 16;81(4):201–8. doi: 10.1136/oemed-2023-109315 (PMC11103336; doi:10.1136/oemed-2023-109315)
Supplement: Supplementary data [file oemed-2023-109315supp001.pdf]

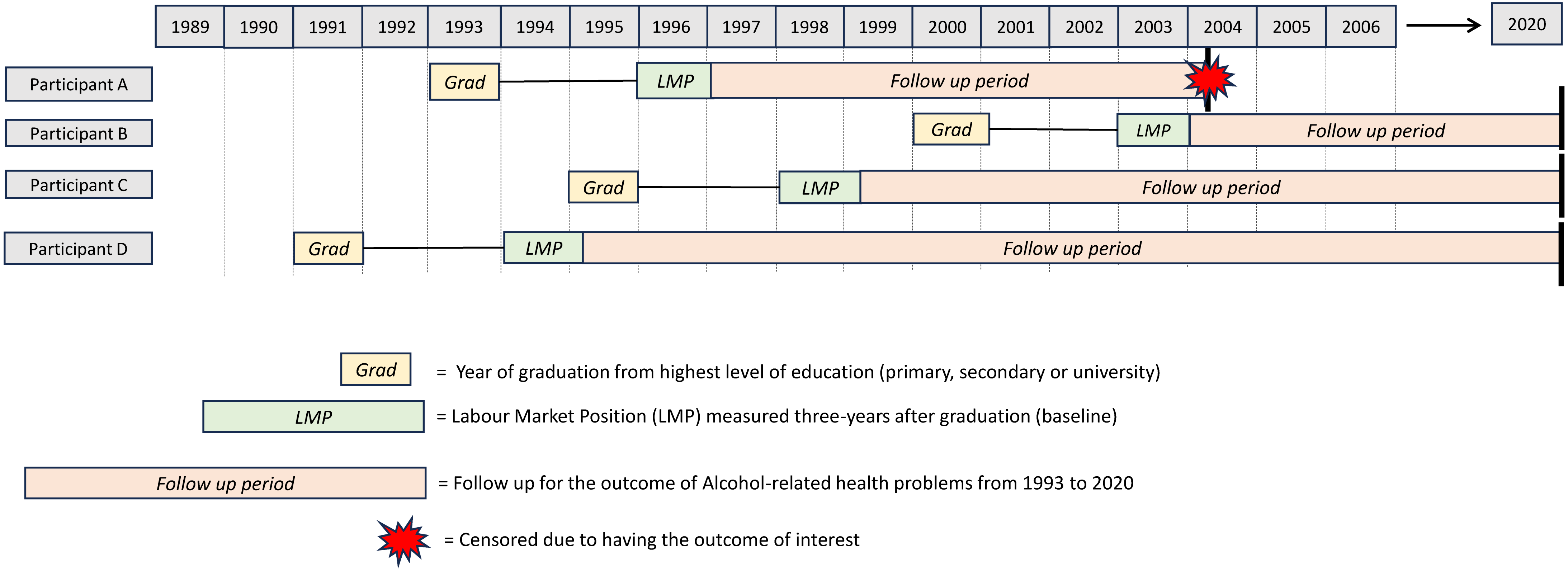

Supplementary figure 1. Study Design: Examples demonstrating the study timeline for measuring year of graduation, labour market position and the follow-up period
